# Obstetric and Perinatal Outcomes after Very Early Preterm Premature Rupture of Membranes (PPROM)-A Retrospective Analysis over the Period 2000–2020

**DOI:** 10.3390/medicina57050469

**Published:** 2021-05-11

**Authors:** Ernesto González-Mesa, Marta Blasco-Alonso, María José Benítez, Cristina Gómez-Muñoz, Lorena Sabonet-Morente, Manuel Gómez-Castellanos, Osmayda Ulloa, Ernesto González-Cazorla, Alberto Puertas-Prieto, Juan Mozas-Moreno, Jesús Jiménez-López, Daniel Lubián-López

**Affiliations:** 1 Surgical Specialities, Biochemistry and Inmunology Department, Malaga University, 29071 Málaga, Spain; egonzalezmesa@gmail.com (E.G.-M.); gonzalezcazorlae@gmail.com (E.G.-C.); 2 Obstetrics and Gynecology Service, Regional University Hospital of Malaga, 29011 Málaga, Spain; martablascoalonso@gmail.com (M.B.-A.); kristmu@hotmail.com (C.G.-M.); lorenasabonet@gmail.com (L.S.-M.); manugocas@hotmail.com (M.G.-C.); osmayda.ulloa.sspa@juntadeandalucia.es (O.U.); jjimenezme35426@gmail.com (J.J.-L.); 3 Obstetrics and Gynecology Service, Virgen de la Victoria University Hospital, 29010 Málaga, Spain; mariajosebenitezmarin@gmail.com; 4 Obstetrics and Gynecology Service, Virgen de las Nieves University Hospital, 18014 Granada, Spain; apuertas51@hotmail.com; 5 Department of Obstetrics and Gynecology, University of Granada, 18016 Granada, Spain; 6 Consortium for Biomedical Research in Epidemiology & Public Health (CIBER Epidemiología y Salud Pública-CIBERESP), 28029 Madrid, Spain; 7 Biohealth Research Institute (Instituto de Investigación Biosanitaria Ibs.GRANADA), 18014 Granada, Spain; 8 Department of Obstetrics and Gynecology, University Hospital of Jerez de la Frontera, Faculty of Medicine, University of Cadiz, 11407 Cadiz, Spain; daniel.lubian@uca.es

**Keywords:** very early PPROM, perinatal mortality, premature birth

## Abstract

*Background and Objectives*: Pre-term premature rupture of membranes (PPROM) responds for one third of preterm births, and it is associated with other complications that increase the risk of maternal or fetal poor outcome. To reduce uncertainty and provide accurate information to patients, the analysis of the large series is of great importance. In order to learn about the evolution over the time of the obstetric and perinatal outcomes in cases of PPROM at, or before, 28 weeks (very early PPROM) managed with an expectant/conservative protocol, we have designed the present study. *Materials and Methods*: We retrospectively studied all cases of very early PPROM attended in Malaga University Regional Hospital from 2000 to 2020. *Results*: Among 119,888 deliveries assisted, 592 cases of PPROM occurred in pregnancies at or before 28 weeks (0.49% of all deliveries, 3.9% of all preterm births and 12.9% of all cases of PPROM). The mean duration of the latency period between PPROM and delivery was 13.5 days (range 0 to 88 days), enlarging over the years. The mean gestational age at delivery was 27 weeks (SD 2.9; range 17–34). The proportion of cesarean deliveries was 52.5%. The overall perinatal mortality rate was 26.5%, decreasing over the period with a significant correlation Pearson’s coefficient −0.128 (*p* < 0.05). *Conclusions*: In the period 2000–2020, there was an improvement in the outcomes of very early PPROM cases and perinatal mortality showed a clear trend to decrease.

## 1. Introduction

Pre-term premature rupture of fetal membranes (PPROM) complicates 2–3% of all pregnancies [1]; responds for one third of all cases of preterm birth [2]; and it is associated with secondary complications, which increase the risk of maternal or fetal poor outcome, like placental abruption, cord prolapse and intraamniotic infections [3]. The etiology of PPROM remains unknown in most cases, and some genetic, environmental, mechanical, microbiological and inflammatory factors have been described [4]. It is accepted that preterm uterine contractions or mechanical distention of fetal membranes increase the risk of PPROM [5]. Also, the prevalence of microbial invasion of the amniotic cavity has been reported in half of the cases of PPROM [6], pointing out the role of microbial involvement; and some genetic predisposition regarding polymorphism of MMP-2 has been described to be associated with a higher rate of PPROM [7].

Most neonatal short- and long-term complications in cases of PPROM are predicted by gestational age at delivery [8,9,10]. The prolongation of pregnancy needs to be considered the primary goal of expectant management when infection is not present [8], especially in very early onsets. In these cases, a careful balance between maternal and neonatal risks is needed since a prolonged latency period between PPROM and delivery improves neonatal outcomes, but it could also increase the risk of chorioamnionitis [11,12]. The perinatal mortality figures associated with very early PPROM (at or before 28 weeks) are high [13,14], but in recent years, some studies have demonstrated that outcomes for neonates delivered following very early PPROM may be better than previously expected [10]. Although the long-term neurodevelopmental outcomes after PPROM will depend on the interaction between gen-environment, and the etiology of some central nervous system injuries that can be found in some children born after very early PPROM remain unclear [8], the short-term benefits of an expectant and conservative management are well established. In fact, we could previously report in our setting [15] an encouraging upward trend in the duration of latency period over the first decade of this century, and a decrease in perinatal mortality associated with PPROM very far from term.

In order to learn about the evolution over the time of the obstetric and perinatal outcomes in cases of very early PPROM managed with an expectant/conservative protocol, we have designed the present study. Our goal was to review perinatal survival in cases of very early PPROM attended in our maternity from 2000 to 2020, analyzing the temporal trends of variables such as length of latency period, g.a. at delivery, mode of delivery, obstetric complications (cord prolapse, chorioamnionitis and placental abruption) and perinatal survival.

## 2. Materials and Methods

### 2.1. Study Design and Patients

We reviewed clinical records for cases of PPROM at or before 28 weeks admitted to our obstetric department between 1 January 2000 and 31 December 2020.

After we obtained institutional authorization, we revised Andalusian case mix (minimum basic data set registry) [16] to identify the records with diagnostic codes of PPROM at or before 28 weeks. Once all the cases were identified, we reviewed the medical records for information concerning the length of latency period, mode of delivery, gestational age and obstetric complications as chorioamnionitis, cord prolapse or placental abruption. The perinatal mortality data were obtained from the perinatal database of Neonatology and Obstetrics Departments, identifying newborns delivered alive and admitted to the intensive care unit until they were discharged or died. This database also included stillbirths. Extended perinatal mortality was considered, i.e., intrauterine fetal demises from 22 weeks g.a. and neonatal deaths until 28th day of life after delivery.

### 2.2. Statistical Analysis

Statistical analysis was performed using IBM SPSS Statistics v24 software. The Chi square test was used to compare qualitative variables, and the *t*-test or ANOVA were used to compare means between groups according to the number of categories of each variable, always after confirming the normality of the distribution. We used Pearson’s coefficient for correlations, and the area under the curve (AUC) analysis to predict the survival probability of fetuses according to the gestational age at diagnosis.

The data were treated statistically as a whole, aggregate per year and per g.a. at PPROM, with the exception of those pregnancies with pre-viable g.a. at PPROM (before 24^0/7^ weeks) that were considered as a distinct group.

### 2.3. Instruments

In the years of study an expectant-conservative management in cases of PPROM was followed. All patients were hospitalized after diagnosis. A complete bed rest regimen was advised in order to avoid amniotic fluid leakage and cord prolapse. We used prophylactic antibiotics (simultaneous regimen of Ampicillin and Erythromycin for one week), a single course of steroids (betamethasone 12 mg) when g.a. > 24^0/7^ weeks, and tocolitycs (atosiban) during 48 h if contractions were noted. After admission amniotic fluid samples were also taken for conventional aerobic cultures (antibiotic regimen was modified according to the bacterial resistances), and leucocytes and Reactive C Protein (CRP) were assessed every 48 h. If clinically stable, women with pre-viable PPROM (the edge of viability [17] was established at 24^0/7^ weeks), could leave the hospital until viability was reached if they desired, following weekly ambulatory leukocytes and CRP controls. Over the 21 years of study, only a few cases underwent ambulatory management until viability and almost all women chose to remain hospitalized.

In the absence of complications either induction of labor or cesarean delivery for obstetric indications were performed in g.a. > 34^0/7^. When PPROM occurred before g.a. 22^0/7^ weeks, the patient could request legal termination.

The amniotic fluid sampling for culture was extended to Mycoplasma, Chlamydia and anaerobes search in 2006. Prophylactic antibiotic regimen was modified according to the bacterial resistances observed in antibiograms [18]. The clinical diagnosis of chorioamnionitis were made according to Gibbs’ clinical and biological criteria [19], and in all cases there was histological confirmation. Gibbs’ clinical criteria for chorioamnionitis included temperature of at least 37.8 °C, and two or more of the following: Maternal tachycardia, fetal tachycardia, uterine tenderness, foul odor of the amniotic fluid and maternal leukocytosis. In cases of suspected chorioamnionitis that did not meet Gibb’s criteria, amniocentesis was performed to confirm intraamniotic infection if low levels of glucose, positive Gram-straining or microbiological cultures were found in the amniotic fluid analysis.

In cases of extremely pre-viable PPROM we included those cases in which patients chose to continue with the pregnancy and therefore the protocol of conservative management was adopted. This means the loss of those other cases in which patients chose the legal termination of pregnancy, where the follow-up was more difficult given that most of the cases were discharged to be sent to specific clinics to undergo termination.

### 2.4. Ethics

The study was conducted in accordance with the Declaration of Helsinki, and the protocol (*ecarpmp*) was approved on 12 July 2016 by the reference Research Ethics Committee.

## 3. Results

**Figure 1:**
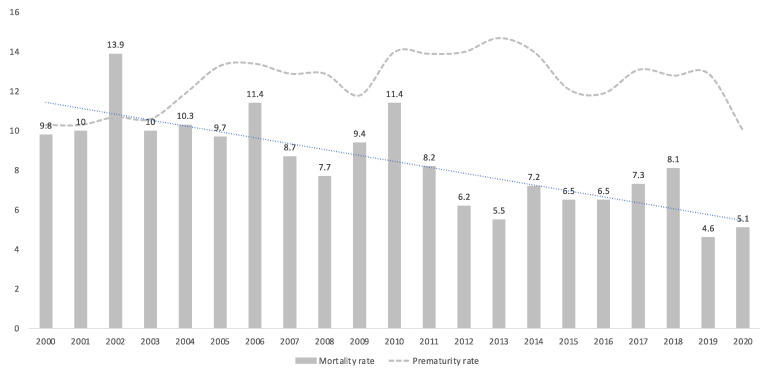
Perinatal mortality (‰) and prematurity rates (%) per year of study.

From 1 January 2000 to 31 December 2020 a total of 119,888 deliveries were assisted in Malaga University Regional Hospital. Among them, 14,931 were under 37 weeks, and 4591 cases of PPROM were diagnosed. These represented 3.8% of all deliveries attended and 30.7% of all preterm births. A total of 592 (0.5% of all deliveries, 3.9% of all preterm births and 12.9% of cases all of PPROM) occurred in pregnancies at, or before, 28 weeks (very early PPROM). The prematurity rates and the perinatal mortality rates from 2000 to 2020 in our maternity are shown in Figure 1.

**Figure 2:**
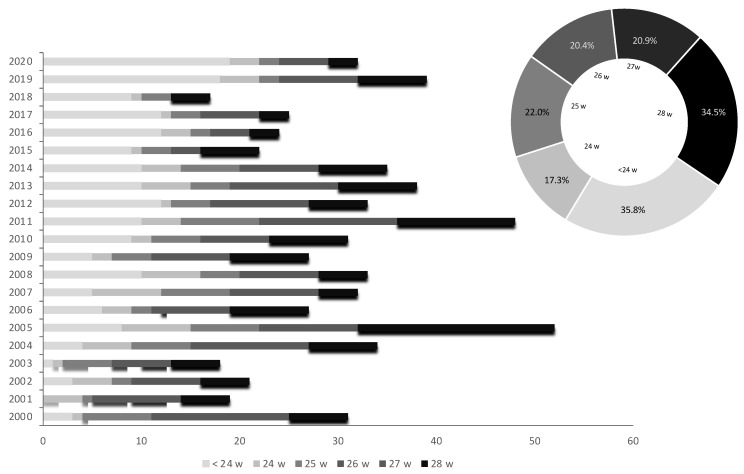
Yearly distribution of the sample according to gestational age at diagnosis of PPROM.

The distributions of the 592 cases of very early PPROM per year and g.a. at diagnosis are shown in Figure 2. Mean maternal age in the period was 31.5 years, with a clear trend to increase over the years (*r* = 0.14, *p* < 0.001), so that mean maternal age was 2.4 years higher in the period 2015–2020 when compared with the first five-years period (32.8 against 30.4 years, *p* < 0.01). We also found a negative correlation between the mean age of the participants and the gestational age at PPROM diagnosis (*r* = −0.12; *p* < 0.01).

**Figure 3:**
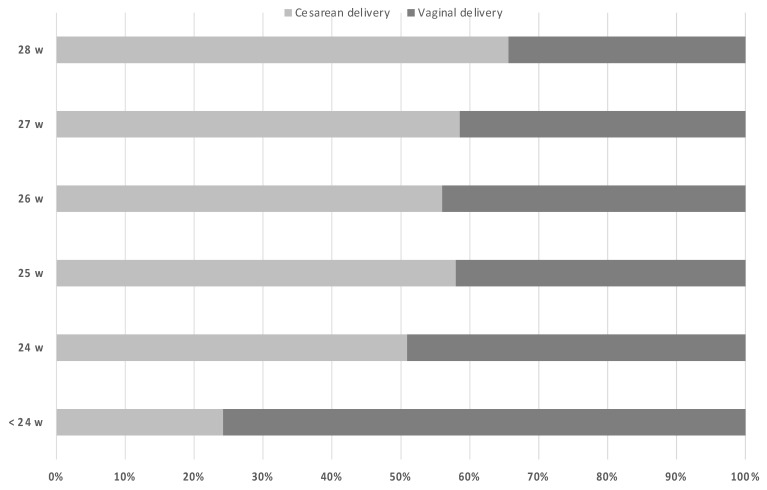
Type of delivery according to gestational age at delivery.

In this population (*n* = 592), 64 placental abruption (10.8%), 112 clinical chorioamnionitis (18.9%) and 9 cord prolapse (1.5%) were diagnosed during hospital admission, so that it was necessary to deliver urgently regardless of g.a. It should be noted that 19 cases (3.2%) were carriers of cervical cerclage and 60 cases (10.1%) needed the use of tocolytics (i.v. atosiban for 48 h). In the pre-viable group, cesarean sections were performed only for maternal indications (severe medical conditions or prior uterine surgery). The proportion of cesarean deliveries was 52.5%. No differences were observed over the years, but cesarean deliveries were significantly more frequent when g.a. at delivery was higher (*p* < 0.001) (Figure 3). We did not find any significant relationship between maternal age and the duration of latency period between PPROM and delivery, the appearance of complications, or the type of delivery.

**Figure 4:**
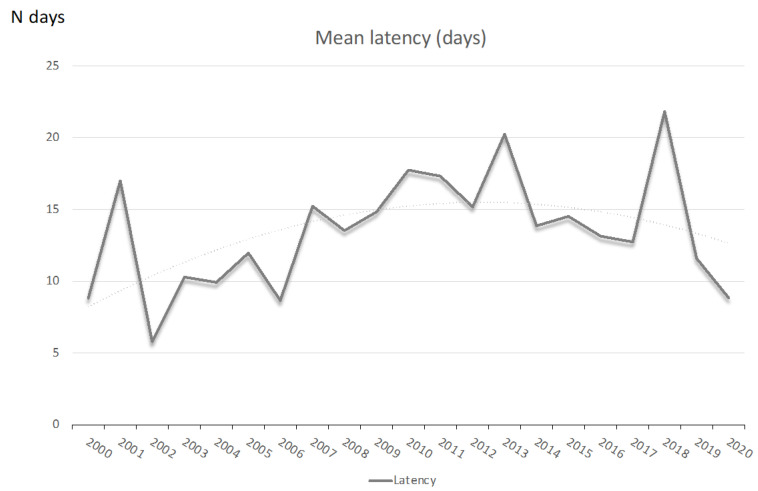
Temporal trend for latency period between PPROM and delivery.

In the very early PPROM population the mean gestational age at delivery was 27 weeks (SD 2.9; range 17–34). The mean duration of latency period between PPROM and delivery was 13.5 days (range 0 to 88 days), enlarging over the study (12.3 days in the period 2000–2010 and 15.1 in the second period, *p* < 0.05), as shown in Figure 4.

**Figure 5:**
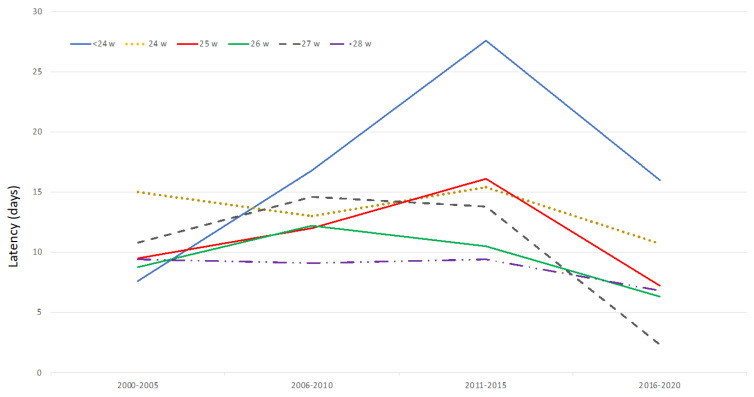
Mean latency period between PPROM and delivery in groups of gestational age at PPROM diagnosis.

Mean latency values between PPROM and delivery for the periods 2000–2005, 2006–2010, 2011–2015 and 2016–2020 were 10.1, 12.8, 16.8, and 12.9, respectively (Figure 5).

We have observed a decreased in the duration of the latency period between PPROM and delivery during the last five-year period. According to g.a. at PPROM diagnosis, a significant reduction of the latency between PPROM and delivery has been found in the group of pre-viable PPROM, in the second half of the last decade (mean difference between the periods 2011–2015 and 2016–2020 = 8.3 days, SD 3.7; *p* < 0.01).

**Figure 6:**
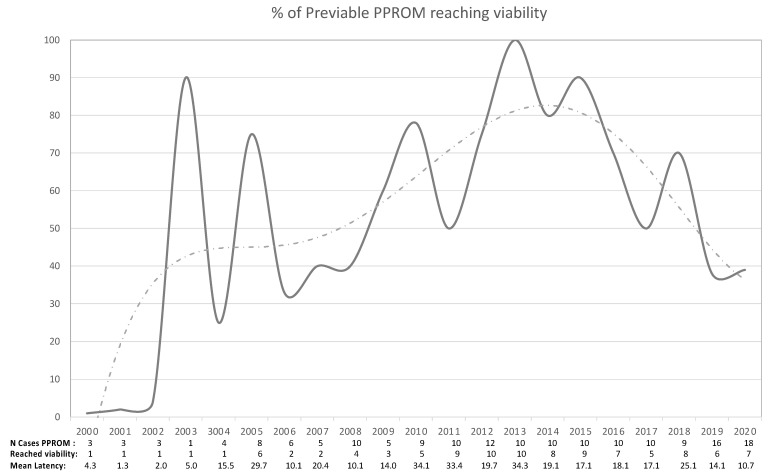
Rate of cases with pre-viable PPROM that reaches viability (gestational age 24 weeks).

A significant correlation between the duration of the latency period between PPROM and delivery and the year of study was found in the period 2000–2010 (Pearson’s coefficient = 0.148; *p* < 0.01), but not within the period from 2011 to 2020. In the group of previable PPROM, a clear trend towards the prolongation of latency between PPROM and delivery was observed since 2005, so that in 2009 and 2010 more than half of patients with PPROM before 24^0/7^ weeks reached viability, and in 2010 one out of three exceeded 28 weeks. This trend decreased during the period 2016–2020, so that in the last year of observation (2020), only the 39.9% of pre-viable PPROM reached viability (7/18), and none of them reached 28 weeks (Figure 6).

**Figure 7:**
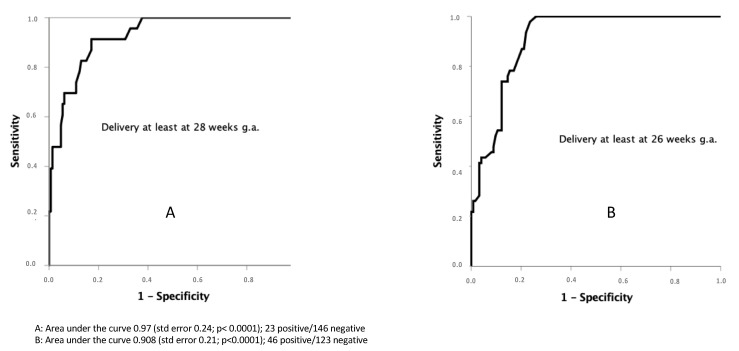
ROC curves for latency duration and delivery over 28 or 26 weeks.

Considering the overall sample of pre-viable PPROM (*n* = 169), at the moment of diagnosis and admission to the hospital the chance to deliver at 28 weeks g.a. was 13.7%. After seven uneventful days following the diagnosis, the probability to deliver at 26 or 28 weeks of g.a. was 54.5%, or 45.9%, respectively, and 76.4% or 62.3% after 14 days of latency (ROC curves at Figure 7).

When pre-viable PPROM occurred before 17, 20 or 22 weeks the probability to deliver over the 28 weeks g.a. was 6%, 26.7%, and 65.8% respectively, and 6.5%, 27.6% and 66.7% to deliver at least at 26 weeks g.a.

**Figure 8:**
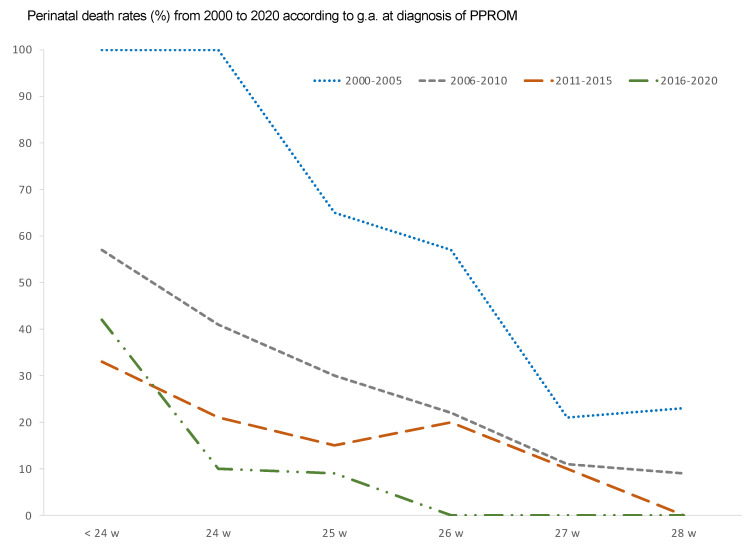
Perinatal mortality rate according to gestational age at PPROM diagnosis.

The overall perinatal mortality rate was 26.5%, decreasing over the period, and showing a significant correlation Pearson’s coefficient −0.128 (*p* < 0.05). Neonatal deaths accounted for 58.7% of the whole perinatal mortality, and fetal mortality was 42.3%. While in the first decade the rate was as high as 30.6%, in the period from 2011 to 2020 it was 22.3% (Figure 8).

**Figure 9:**
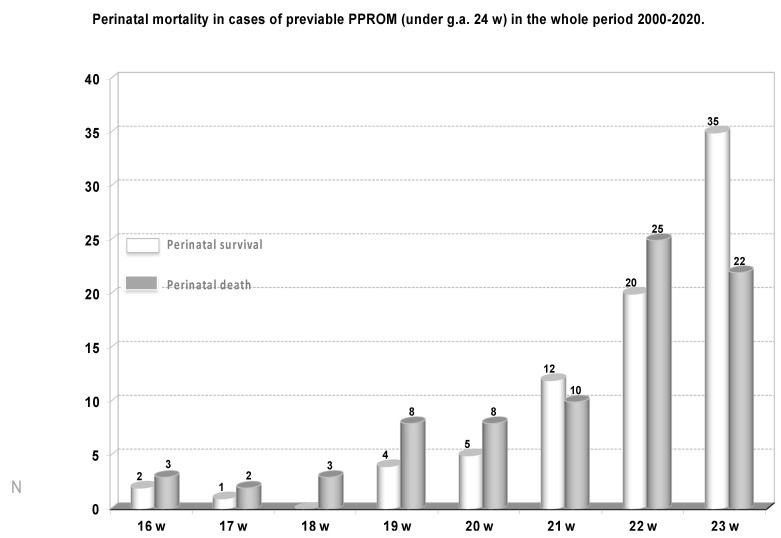
Perinatal mortality and survival in cases of pre-viable PPROM.

The duration of the latency period from diagnosis until delivery correlated significantly with perinatal mortality (Pearson’s coefficient 0.234; *p* < 0.0001). However, the perinatal mortality in the sample was high regardless g.a. at diagnosis of PPROM, reaching 46.2% (75/162) in the group of previable pregnancies (Figure 9), and 37.8 (25/66), 25.3% (21/83), 22.6% (17/75), 10.6% (8/75) and 7.75% (9/116) in cases of PPROM at 24, 25, 26, 27, and 28 weeks, respectively.

## 4. Discussion

This paper shows the obstetric outcomes and perinatal mortality rates in a large sample of pregnancies complicated with very early PPROM admitted at Malaga University Regional Hospital during the period 2000–2020. We confirmed the decreasing trend in the perinatal mortality rates associated with this complication, as well as the prolongation of the latency period until delivery over the years. However, some fluctuations have been observed throughout the whole period, but a clear trend towards the improvement is ensured, we must be aware of the increase in the mortality figures found in the last five years, specifically in the group of pre-viable PPROM in which perinatal deaths reached 42%, nine points over the rate observed in the period 2011–2015. This increase is mainly due to the number of deaths registered during the last two years of the period greater than those observed before (28% in 2017, 20% in 2018, 35% in 2019 and 36.5% in 2020). We do not have a clear explanation for this increase because there were no changes in the management of these pregnancies, except for some necessary changes in the staff who provided care in the obstetric ward, and for all the organizational changes in the obstetric ward due to the COVID-19 pandemic during 2020. The pandemic produced some changes in the obstetric care, due to the need for hospital beds to admit patients with Covid-19, and to the effects of the pandemic on the hospital staff. These changes could have influenced the results.

At present, it is clear enough that the use of prophylactic antibiotics is beneficial for newborns and their mothers. It has been shown a prolongation of the latency period, a decrease in diagnoses of chorioamnionitis, neonatal respiratory distress and sepsis [20,21]. In our Hospital the ORACLE II study provided consistency in antibiotic treatments for PPROM, subjected to some variability until 2003 [22]. Benefits arising the use of corticosteroids to accelerate fetal lung maturation and reduce the risk of necrotizing enterocolitis and intraventricular hemorrhage cases of PPROM over 24^0/7^ weeks are also important [23,24,25]. In our series, the latency period has been progressively extended over the years. Actions, such as the homogeneity in antibiotic treatment promotion, periodical sampling for amniotic fluid cultures and additional antibiotic courses in cases of isolation of pathogens according to the antibiogram, have contributed to increase the latency period until delivery. This reduces the effect of extreme prematurity in the figures perinatal mortality associated to PPROM.

When PPROM complicates the pregnancy far from term, at pre-viable g.a., obstetricians have to deal with the uncertainty that this condition entails. The lack of prospective, randomized and controlled trials, and the variety of variables influencing neonatal and maternal outcomes in cases of PPROM, means that clinicians have to deliver information based on the results of retrospective studies with a limited number of cases. In order to provide accurate information to these patients, the analysis of the results of large local series is of great importance. In our experience, when the information includes encouraging data, in terms of latency prolongation and survival rate of preterm newborns, women often accept an active/expectant management with hospitalization, bed rest, antibiotics and corticosteroids from g.a. 24^0/7^ weeks. On the other hand, when the information is limited to providing perinatal mortality and morbidity rates, women usually request legal termination of the pregnancy.

In our maternity, the diagnosis of PPROM involves the patient’s institutionalization in order to facilitate early diagnosis of possible chorioamniotic infection. We also recommend bed rest, at least during the first days after diagnosis, to prevent loss of amniotic fluid and cord prolapse. Although there is not enough evidence of the benefits of hospitalization, bed rest [26,27] or amnioinfusion in patients with pre-viable PPROM [28,29,30], most of the women with pre-viable PPROM opt for admission in the obstetric ward and bed rest. In these cases, weight-adjusted daily doses of low-molecular-weight heparin are included in the treatment.

All the data included in this paper provide valuable information to advance a forecast about perinatal outcome after extremely PPROM diagnosis, being the largest sample (592 cases) of very early PPROM that has been published in our country. However, its design has some limitations derived from the retrospective review of the clinical information and the accuracy of the records. Over the years of study, some important changes in the information and recording systems made it very difficult to gather homogeneous data sets on sociodemographic and non-clinical variables, and only maternal age was available for analysis as a cofactor. Also, this series only includes the cases of women with pre-viable PPROM that opt for an active/expectant management, and not those who request legal termination, whose records were not available.

## 5. Conclusions

We have confirmed a decreasing trend in the perinatal mortality rates associated with PPROM, and a prolongation of the latency period until delivery over the years of study.

## Data Availability

No new data were created or analyzed in this study. Data sharing is not applicable to this article.
